# Deep Dilated Convolutional Neural Network for Crowd Density Image Classification with Dataset Augmentation for Hajj Pilgrimage

**DOI:** 10.3390/s22145102

**Published:** 2022-07-07

**Authors:** Roman Bhuiyan, Junaidi Abdullah, Noramiza Hashim, Fahmid Al Farid, Wan Noorshahida Mohd Isa, Jia Uddin, Norra Abdullah

**Affiliations:** 1 Faculty of Computing and Informatics, Multimedia University, Persiaran Multimedia, Cyberjaya 63100, Malaysia; junaidi.abdullah@mmu.edu.my (J.A.); noramiza.hashim@mmu.edu.my (N.H.); fahmid.farid@gmail.com (F.A.F.); wan.noorshahida.isa@mmu.edu.my (W.N.M.I.); 2 AI and Big Data Department, Endicott College, Woosong University, Daejeon 300-718, Korea; jia.uddin@wsu.ac.kr; 3 WSA Venture Australia (M) Sdn Bhd, Cyberjaya 63100, Malaysia; norra@wsaventure.com

**Keywords:** crowd density classification, deep augmentation, morphological operation, FCNN, Hajj-Crowd dataset

## Abstract

Almost two million Muslim pilgrims from all around the globe visit Mecca each year to conduct Hajj. Each year, the number of pilgrims grows, creating worries about how to handle such large crowds and avoid unpleasant accidents or crowd congestion catastrophes. In this paper, we introduced deep Hajj crowd dilated convolutional neural network (DHCDCNNet) for crowd density analysis. This research also presents augmentation technique to create additional dataset based on the hajj pilgrimage scenario. We utilized a single framework to extract both high-level and low-level features. For creating additional dataset we divide the process of images augmentation into two routes. In the first route, we utilized magnitude extraction followed by the polar magnitude. In the second route, we performed morphological operation followed by transforming the image into skeleton. This paper presented a solution to the challenge of measuring crowd density using a surveillance camera pointed at a distance. An FCNN-based technique for crowd analysis is included in the proposed methodology, particularly for classifying crowd density. There are several obstacles in video analysis when there are a large number of pilgrims moving around the tawaf area, with densities of between 7 and 8 per square meter. The proposed DHCDCNNet method has achieved accuracy of 97%, 89% and 100% for the JHU-CROWD dataset, the UCSD dataset and the proposed Hajj-Crowd dataset, respectively. The proposed Hajj-Crowd dataset, the UCSD dataset, and the JHU-CROW dataset all had accuracy of 98%, 97% and 97%, respectively, using the VGGNet approach. Using the ResNet50 approach, the proposed Hajj-Crowd dataset, the UCSD dataset, and the JHU-CROW dataset all had an accuracy of 99%, 91% and 97%, respectively.

## 1. Introduction

There is a growing demand for computer models capable of evaluating very dense crowds using video feeds from security cameras because of the issues associated with ineffective crowd management, such as crowd crushes and blockades. An automated technique for assessing crowd density relies heavily on crowd analysis. This includes determining the size of the crowd and the density of the crowd’s spread throughout the entire gathering place. Advance warnings and the prevention of severe crowd crushes can be prevented by identifying places with crowd densities exceeding the safety limit. This also helps to quantify the significance of the event and to manage logistics and infrastructure for the gathering more effectively by estimating crowd size.

In this article, we propose a technique based on deep augmentation for forecasting crowd density and performing crowd analysis on still images. Crowd analysis poses a variety of challenges in heavily inhabited areas. Crowd images with a high density of people suffer from extreme occlusion, rendering typical face and person detectors useless. Crowd images can be captured from a variety of angles, introducing perspective difficulties. Due to the non-uniform scaling of the crowd, the estimating model must be scale invariant in the event of large-scale changes. Additionally, unlike with other visual challenges, annotating photographs of large crowds is a tough task. This precludes the possibility of large-scale crowd analysis datasets and limits the amount of available training data for learning-based systems.

Hand-crafted image features (SIFT [1], HOG and others [2]) often fall short of providing resilience to occlusion and large-scale variation issues. They also used augmentation techniques such as morphological image processing in some works without using deep learning methods [3,4]. Instead of that, the technique for crowd analysis is based on in-depth learnt features through fully convolutional neural networks(FCNN) gives better performance.

In CSRNet, Li et al. [5] show via a test that MCNN [6] outperforms a more deep, conventional system. The research focuses on a two-level deep growth-based aberration approach, which includes estimate in a highly obstructed scene. Unlike other circuitous techniques used in computer vision challenges, the first phase is to expand the dataset so that a profound system may detach both high- and low-level highlights at the following stage of highlight extraction. This article developed an approach that takes the complete image as information and computes the final tally directly. In the midst of this, there is one more expansionary era. Each image is divided into patches, and these patches are managed by a sophisticated method that eliminates the fix highlights associated with their tally. Finally, the relapse system leverages these neighborhood checks of patches to establish a mapping between highlights and tally in order to forecast the image’s global total. Other contributing approaches are also employed to more accurately estimate the crowd size and density. As in CNN-MRF [7] we use Markov Random Field to smooth the counting of neighboring overlapping patches.

By merging a shallow and a deep convolutional architecture, we overcome the issue of size variation in crowd images. Additionally, we significantly enrich the data by sampling patches from the multi-scale image representation to ensure that the system is scale-independent. On the tough UCSD dataset [7], the approach displays state-of-the-art performance.

In this research, we proposed a method for crowd density analysis using fully convolutional neural networks (FCNNs) with an augmented dataset for the Hajj pilgrimage. There is no standard dataset based on Hajj pilgrimages crowd analysis. There is a demand to develop a novel dataset based on Hajj crowd.

The key contributions are summarized below:

- To create a Hajj crowd deep convolution neural network with configurable dilation rates and a number of convolution kernels to overcome the data movement issue introduced by the dilated convolution architecture.
- For accurate crowd comprehension in severely congested crowd environments, we offer a unique DHCDCNNet architecture based on Hajj crowd DCNNs.
- We have created a novel Hajj-Crowd dataset based on a set of images by using augmentation techniques.

The rest of the paper is arranged in this way: Section 3 presents the proposed method; Section 4 presents the implementation; Section 5 shows the details result evaluation and comparison and Section 6 present the conclusion.

## 2. Related Work

In the past few years, crowd comprehension has garnered considerable interest. There are several techniques for crowd comprehension, which may be classified as traditional or deep learning. Additionally, there are three types of conventional methods: (1) detection-based on methods; (2) approaches based on regression; and (3) methods based on density estimation. Thus far, deep learning techniques have dominated the field of crowd comprehension. We will briefly discuss some crowd analysis techniques in this section.

### 2.1. Conventional Techniques

#### 2.1.1. Detection-Based Method

Detection was the first step in crowd understanding, with a sliding window used to distinguish pedestrians and count the number of people in crowd images [8]. Furthermore, detection methods are divided into two categories: whole-body detection and partial-body detection (e.g., head and shoulder). In general, these strategies start by extracting features from crowd images (e.g., Haar wavelet [9], edgelet [10]), and HOG (histogram-oriented gradients [1]) within a sliding window and then categorizing those features using a pre-trained classifier (e.g., support vector machines [8,11]). Systems based on part-body detection perform marginally better than approaches based on full-body identification when it comes to occlusion in crowds. While techniques based on part-body identification may alleviate some of the difficulties associated with crowd occlusions, they continue to perform poorly in tightly packed crowd settings with a complex background.

#### 2.1.2. Approaches Based on Regression

The association between characteristics and the quantity of individuals in image patches is investigated using regression-based approaches for crowd comprehension. In regression-based techniques, extracting features (e.g., foreground features, edge features, texture and gradient characteristics) from crowd images and applying regression algorithms to establish the mapping between features and the number of people are often three important processes (e.g., linear regression [12], piecewise linear regression [13], and ridge regression [13]). It is possible to reduce the opacity and clutter of crowd images using regression-based algorithms, but they do not take into account the spatial information included in the images.

#### 2.1.3. Approaches Based on Density Estimate

Methodologies that employ density estimates instead of regression concentrate on creating density maps for crowd images. It was postulated by Lempiitsky et al. [14] that a linear mapping between the properties of local patches and their corresponding item density maps may be learned by including spatial information. Due to the difficulty of discovering an exact relationship between the features of local patches and the relevant item density map, Pham et al. [15] used random forest regression to discover a nonlinear relationship. It was proposed by Wang and Zou [16] that each picture patch’s embedding in a subspace-based approach be covered by the image.

#### 2.1.4. Deep Learning Based Approaches

As deep learning has matured, many CNN-based models for crowd comprehension in crowded contexts have been developed. With these techniques, background noise, occlusions, and perspective distortion may all be efficiently reduced, while the mean absolute error (MAE) is significantly reduced compared to past methods [6,17,18,19]. Using layered boosting and selective sampling, Walach et al. [18] developed a CNN-based approach that improves crowd counting accuracy. For example, Shang et al. [6]’s end-to-end CNN architecture takes a complete crowd picture as an input and simultaneously learns both local and global counts. By combining shallow and deep networks, the CrowdNet dual-column architecture by Boominathan et al. [17] is able to gather semantic information at both a high and low level. CrowdNet’s first 13 layers are used as a deep network in VGG-16, resulting in poor performance [5]. To deal with multi-scale contextual information in crowd images, Zhu, Zhang, and Liu devised MCNN as an answer to the image classification assignment in [20]. It is, however, hard to extract all of the model’s features, resulting in low-quality density maps because of the model’s characteristics. Switching-CNN was proposed by Deepak et al. [18] to forecast the density map based on crowd density, which is MCNN-based. The classifier decides which convolutional neural network is used to predict the density map. A contextual pyramid convolutional neural network, as developed by Sindagi et al. [21], integrates global and local contextual information to produce high-quality density maps (CPCNN). Contextual data are gathered as an afterthought in both Switching-CNN and CP-CNN, rather than being used to improve density map quality right away. Using dilated convolutional layers, CSRNet, created by Li et al. [5], is a crowd interpretation system that can extract more particular characteristics from crowded situations. As a consequence of the “gridding” problem in the dilated convolution framework, the population density in densely populated regions was underestimated. For example, an attention-injective deformable convolutional neural network (ADCrowdNet) developed by Liu et al., for example, combines a visual attention mechanism with a multi-scale deformation convolution algorithm for crowd understanding in densely packed situations. Confidence maps are used to adaptively evaluate individual importance in a CAT-CNN for crowd comprehension developed by Chen et al. [22]. However, because these systems require a significant increase in computing resources, training them is challenging. It is in this study that we provide DMDCNet, a simple yet efficient end-to-end framework for obtaining high-quality density maps.

#### 2.1.5. CNN-Based Approaches

A deep convolutional network is used in CNN-based techniques to forecast the crowd density map and count. Zhang et al. [23] pioneered the use of a multicolumn architecture (MCNN) to analyze and count crowds. The presented network used three simultaneous convolutional networks of varying sizes to extract information at various scales. Shang et al. [20] provided a technique that accepts the picture input in its entirety and produces the final count of people in that image without requiring any patch-based training. He extracted high-level deep features using a pre-trained GoogLeNet, a local count using an LSTM [24], and a final count using fully connected layers. Additionally, it is obvious that techniques based on CNN surpass all prior conventional approaches outlined before.

Sam et al. [18] offered a Switching CNN, whereas Sindagi et al. [24] presented a Contextual pyramid CNN. Both proposed architectures are based on MCNN (Multi-segment neural networks) and a thickness level classifier. The disadvantages of these types of systems are that they need more preparation time due to the multicolumn CNN architecture. Because the lack of parameters reduces the system’s accuracy, while the presence of parameters suggests that the arrangement requires more preparation time. Taking these constraints into account in our approach, we present a deep growth-based technique that does not need a multi-section CNN design and also requires less preparation time. This methodology’s colossal commitment is as follows:

1. To create a novel two-level data augmentation approach that extracts low- and high-level features concurrently, reducing the training time of complex networks.
2. To obtain a more accurate crowd estimate for densely populated scenes, certain pre-trained models and a basic regression network are used.

## 3. Proposed Method

### 3.1. DHCDCNNet

**Figure 1:**
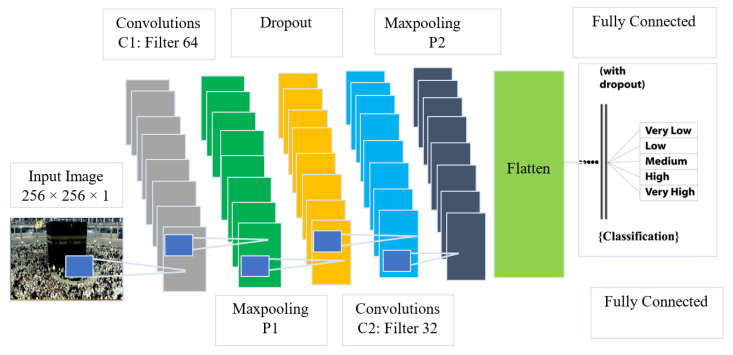
The proposed architecture of DHCDCNNet.

Our research objective is to offer a pure, fully convolutional network architecture for extracting more comprehensive features and gathering all of the data required to construct high-quality density estimates and perform exact crowd analysis. The next parts describe the DHCDCNNet network design and the training technique that goes with it. Figure 1 shows the proposed DHCDCNNet architecture.

As indicated, the DHCDCNNet architecture is as follows: The DHCDCNNet has ten layers, starting with the input layer and concluding with two convolutional layers (CLs). The architecture is comprised of two pooling layers (PLs), two dropout layers (DLs), two fully connected layers (FCLs), and a single output layer. The size of the input layer is specified by SGI (256 × 256 × 1). A kernel size of 5 × 5 was utilized to decrease the number of parameters and enhance training efficiency. The CL1 and CL2 each include 64 and 32 filters. The PL2 sample size is smaller than the CL1 size. The FCL1 translates the CL2’s feature maps to a one-dimensional format. The FCL2 allows the final layer to classify the input data by its intended purpose. The valid convolution technique utilized in this neural architecture ensures that the size of the feature maps remains constant. Additionally, the two dropout layers allow the network to generalize data, hence reducing over teaching [25,26]. As previously mentioned, the backward propagation stage is used to train the neural network (BPS). The basic goal of network training is to reduce objective function error via the BPS update of weights and biases. During the training stage, a deep learning rate is considered to determine the DCNN structure. This deep learning strategy enhances the efficiency of the neural network and prevents it from converging to a local minimum. Additionally, an adaptive moment estimation technique (Adam) is recommended for updating the DCNN’s weights [27]. Adam uses a non-stationary root-mean-square propagation (RMSProp) technique to combine the advantages of deep gradient algorithms (AdaGrad) for dealing with sparse gradients. In each update, Adam keeps track of the gradient’s exponential moving average (EMA) and its square, which are related as follows:

(1) $$w=w-\alpha \frac{B_{mt_{1}}}{\sqrt{B_{mt_{2}}+\varepsilon }}$$

(2) $$B_{mt_{1}}=\beta_{1}B_{mt_{1}-1}+\left(1-\beta_{1}\right)\frac{\partial }{\partial w}\cos t\left(w\right)\;\;\mathrm{here}\;\beta_{1}\approx 1$$

(3) $$B_{mt_{2}}=\beta_{1}B_{mt_{2}-1}+\left(1-\beta_{2}\right)\frac{\partial^{2}}{\partial w^{2}}\cos t\left(w\right)\;\;\mathrm{here}\;\beta_{2}\approx 1$$

where is the positive scalar step size *w* and α is the weight parameter. $B_{mt_{1}}$ and $B_{mt_{2}}$ are the first and second moment bias corrections, respectively, $\beta_{1},\beta_{2}$ are the decay rates. The step size α and decay rates $\beta_{1},\beta_{2}$ are both small, as shown by Equations (2) and (3). As a consequence, the weight update approach in Equation (1) yields a nearly optimal201learning rate choice [27]. As a result, DHCDCNNet is used to refer to the final structure, which is made up of the CNN, the deep learning rate, and Adam in this study. Finally, the hyperparameters (i.e., dropout rate, learning rate, momentum, number of epochs, and batch size) of the recommended architectures are optimized using a grid search-based 5-fold Cross Validation (5-CV). Table 1 shows the proposed DHCDCNNet in detail, along with layer specifications.

### 3.2. Deep Augmentation

Because the group datasets are limited, there is a specified number of preparation tests, and when deep learning algorithms are used, these datasets are not sufficient to create a deep system. When we apply deep learning to these datasets, the outputs are less viable, but more so than they should be. Along these lines, we offer a two-level deep expansion-based approach for group verification, which enables our deep system to deal with the problem of a missing dataset, while also requiring fewer calculation parameters, which reduces the system’s preparation time. We use a different technique for augmenting the training dataset than is typical for augmentation. We use two techniques: first, we send the image as a whole in the form of two-dimensional vectors; second, we calculate the magnitude and angular components of the image; and last, we utilize the magnitude component of the image as an enhanced image for training our network. The magnitude and angle of a two-dimensional vector (x(J),y(J)) may be determined as follows:

(4) $$\mathrm{magnitude}\left(J\right)=\sqrt{x{\left(J\right)}^{2}+y{\left(J\right)}^{2}}$$

(5) angle(J)=atan2(y(J),x(J))[.180/π]

**Figure 2:**
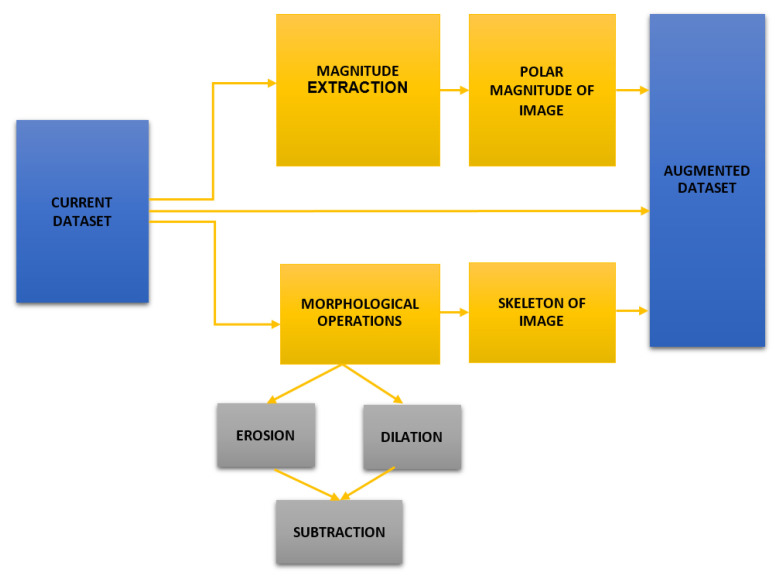
Proposed Augmentation Techniques.

**Figure 3:**
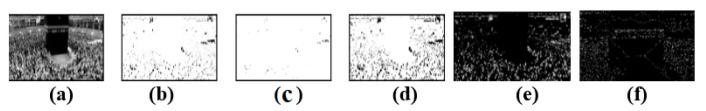
Output of the Augmentation: (**a**) Input Image (**b**) Mask (**c**) Dilation (**d**) Erosion (**e**) Polar Magnitude and (**f**) Skeleton of Image.

Following that, we compute the skeleton of each sample so that our deep network can simply extract the low-level characteristics. The image’s skeleton is calculated by performing the following morphological operations on it: Erosion, Dilation, and Subtraction. Erosion essentially removes the outermost layer of pixels from a structure, while dilation adds an additional layer of pixels. Then, we subtract these two values to get the image’s skeleton. Both of these strategies are applied to all training image samples using Python’s open-cv package. The augmentation procedure is shown in Figure 2. Figure 3 illustrates the outcomes of applying these augmentations to the original dataset.

### 3.3. Dilated Convolution

**Figure 4:**
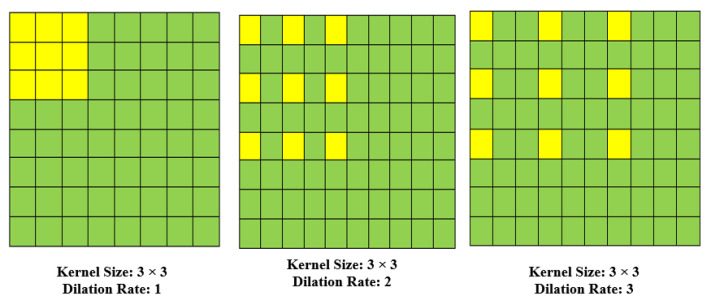
The dilation of rates 1, 2 or 3 with 3 × 3 convolution kernels.

Our design is built around the dilated convolutional layer. In two dimensions, a dilated convolution may be defined as follows:

(6) $$y\left(m,n\right)=\sum_{i=1}^{M}\sum_{j=1}^{N}x\left(m+r\times i,n+r\times j\right)w\left(i,j\right)$$

y(m,n) is the output of a dilated convolution operation using an input x(m,n) and a filter (wIj) with length and width equal to *M* and *N*. *r* represents the rate of dilatation. A dilated convolution becomes a normal convolution when *r* equals one. Dilated convolutional layers have been shown to greatly improve segmentation accuracy [11,28,29] making them a viable alternative to pooling layers. Invariance and overfitting are generally prevented by using pools of layers (for example, maximum and average pooling). However, pooling layers has a significant impact on spatial resolution, and so the spatial information contained in feature maps is obliterated. While deconvolutional layers [22,30] are beneficial in terms of information loss prevention, their increased complexity and execution time may be too long in certain applications. Dilated convolution is a superior choice since it alternates between pooling and convolutional layers while employing sparse kernels, making it a more efficient method of computing (as illustrated in Figure 3). It is possible to enhance the receptive field without increasing the number of parameters or the amount of computation required by using this feature. With a dilated stride *r*, a small-size kernel with a *k* × *k* filter is extended to k+(k−1) with a k×k filter (r−1). As a consequence, it permits flexible aggregation of contextual data at many scales while preserving the same resolution. In Figure 4, a normal convolution results in a receptive field of 3 × 3, but two dilated convolutions result in receptive fields of 5 × 5 and 7 × 7, respectively. Dilated convolution definitely beats the method of convolution + pooling + deconvolution in terms of retaining the resolution of a feature map. The input is a crowd image, which is processed separately in two ways to create a similar-sized output. The first strategy takes a factor of two samples from the input and then sends it to a convolutional layer using a 3 × 3 Sobel kernel. Because the resultant feature map is just half the size of the original input, the deconvolutional layer must increase the sampling rate (bilinear interpolation). On the other hand, dilated convolution may be used to turn the same 3 × 3 Sobel kernel into a dilated kernel with a stride factor of 2 using the same 3 × 3 Sobel kernel. The output has the same physical dimensions as the input (meaning pooling and deconvolutional layers are not required).

Dilated convolution is a method for increasing the size of the kernel (input) by introducing gaps between its successive parts. In simplest words, it is similar to convolution, except that it employs pixel skipping to cover a greater region of the input. Convolution, on the other hand, is the straightforward application of a filter to an input that results in activation. Repeated application of the same filter creates a map of activation’s known as a “feature map”, which indicates the location and strength of a feature in an input, such as an image.

### 3.4. FCNN Network Configuration

Crowd analysis is essentially an interdisciplinary field, including scientists, psychologists, biologists, public security personnel, and specialists in computer vision. In the past few years, computer vision has gained prominence in the area of deep learning. The fully convolutional neural network (FCNN), a very sophisticated deep learning model for grid-style data such as photos, is one of the most powerful deep learning models available. This approach benefits from the use of convulsions to aid in neuronal growth and picture categorization. FCNN is a neural network approach that largely depends on convolutional, polling, and fully connected layers. The picture is represented and the function mapping is computed using the convolutional layer. A convolutional layer, which is composed of a sequence of mathematical processes, is critical in FCNN. After each layer, a polling layer was added to limit the resolution of the function mapping. Typically, a pool layer is used in conjunction with a sampling strategy to lower the spatial dimension in order to identify and remove parameters with the least amount of distortion and function map alteration. Following polling layer sampling, features derived from convolution layers were developed, as well as such characteristics. A completely connected layer of substrates “flattens” the networks that serve as the input for the subsequent layer. Additionally, two neighboring layers contain neurons that are strongly coupled to one another.

### 3.5. Hajj-Crowd Dataset Collection

This section discusses the proposed HAJJ-Crowd dataset from two perspectives: data capture and specification definition and Dataset Construction using Morphological Operations.

(1) Data Capture and Specification Definition

HAJJ-crowd dataset were taken from the YouTube live broadcast in Mecca Hajj from 2015 to 2019. Accordingly, in certain communities around Kaaba (Tawaf region), 27,000 images and 25 video sequences were shot, including some typical crowd scenarios, including touching the black stone in the Kaaba area. The Hajj crowd dataset is a large-scale crowd density dataset. It includes 21,600 training images and 5400 test images with the same resolution (1914 × 922). The proposed method outperforms the state-of-the-art method in the context of a new dataset (Name HAJJ-Crowd dataset).

(2) Dataset Construction using Morphological Operations

**Figure 5:**
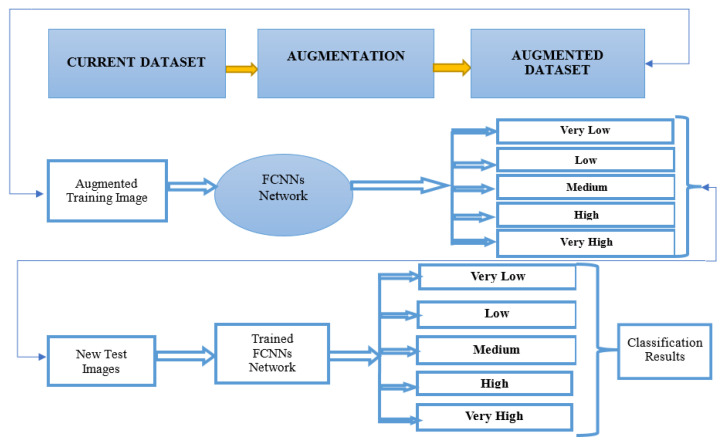
Model of Crowd Density Classification.

For this experiment we need the augmented dataset. Firstly, we have used our current Hajj-Crowd dataset. Secondly, we have used a few augmentation techniques for getting augmented dataset such as Erosion, Dilation, and Subtraction. Finally, after completing the morphological operations we got the augmented dataset. Figure 5 shows the augmented dataset process.

## 4. Implementation

### 4.1. Crowd Analysis Training Method

The training method for crowd density is shown in Figure 5. For the training phase, we utilized an accurate density-labeled image from the previous step to identify the five training classes using fully convolutional neural networks (FCNNs). We train the DHCDCNNet in a straightforward way as an end-to-end structure. Using a well-trained FCNN, the top 10 convolutional layers are tuned. The subsequent layers’ initial values are derived from a Gaussian initialization with a standard deviation of 0.01. Stochastic gradient descent (SGD) is utilized for training with a predefined learning rate of 1 × 10^−6^. Additionally, we measure the disagreement between ground facts using the Euclidean distance. The loss function is as follows:

(7) $$L\left(\Theta \right)=\frac{1}{2N}\sum_{i=1}^{N}{∥Z\left(X_{i};\Theta \right)-Z_{i}^{GT}∥}_{2}^{2}$$

where *N* is the batch size of the training set and $Z\left(X_{i};\Theta \right)$ denotes the output produced by DHCDCNNet using the parameters indicated Θ. $X_{i}$ denotes the input image, whereas $Z_{i}^{GT}$ denotes the input image’s ground truth result.

### 4.2. Crowd Density Process (TESTING)

Figure 5 illustrates the crowd analysis density testing process using FCNNs. First, we prepared a new test image dataset. We then passed the full set of images for testing. Second, we tested five classes using the FCNNs. Finally, we obtained classification results for the five classes.

### 4.3. Dataset Comparison

Our Hajj-Crowd dataset is based on five classes. The five classes are: very low, low, medium, high, and very high. For our experiment, we used another two datasets, the UCSD and JHU-CROWD datasets. However, in the UCSD and JHU-CROWD datasets, they were never divided into different classes. For our evaluation, we have divided five classes manually. Table 2 presents a dataset comparison with current public datasets.

#### 4.3.1. JHU-CROWD

The crowd analysis dataset for JHU-CROWD contains 4250 images (with an average resolution of 1430 × 910) taken under a variety of situations and in a variety of geographic places [32]. Our technique is reviewed and compared to previous research, with the findings summarized in the Section 5. This dataset has 1.51 million dots, an average of 346 dots per image, and a maximum of 25 K dots.

#### 4.3.2. The UCSD Dataset

The UCSD dataset [13] has 2500 security camera frames. These scenes include a sparse throng of between 11 and 46 people per images. Due to the constant and modest resolution of each frame (238 × 158), it is difficult to build a high-quality density image after many pooling procedures. As a result, we pre-process the frames by resizing them to 952 × 632 using bilinear interpolation. According to [13], we take frames 2000 as the training set and the remaining frames as the testing set.

#### 4.3.3. Hajj-Crowd Dataset

Because there are no standard datasets available in the literature at the time of writing, a labelled image sequence is included in this paper. The proposed HAJJ-crowd dataset was collected from live television broadcasts via YouTube of the Mecca Hajj 2019. All of the images depict pilgrims performing tawaf around the magnificent Kaaba. Tawaf involves walking around the Kaaba seven times. The moving process begins in the opposite direction of the clock. The video frames have been extracted and saved as .jpg files for future examination. The dataset contains a total of 27,000 crowd images. As a result, 27,000 images and 25 film sequences are captured in several populous areas surrounding Kaaba (Tawaf region), with some typical crowd scenarios, such as touching a black stone in the Kaaba region. All images have a resolution 1920 × 920 HD and videos have a resolution 1080p.

## 5. Result Evaluation and Comparison

### 5.1. Experiment 1(FCNN)

**Figure 6:**
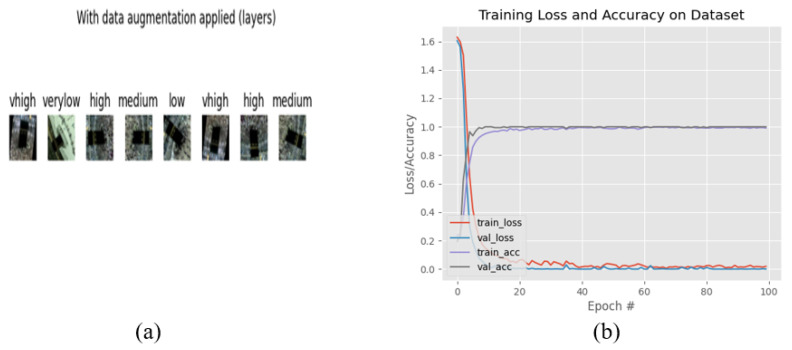
Graphical Representations: (**a**) Data Augmentation applied (**b**) Five Classes Results.

The UCSD dataset contains a total of 2500 images, and each class contains 500 images. The JHU-CROWD dataset consists of 4000 images, and each class has 800 images. For training we have used 80% and testing 20%, respectively, for both of the datasets. All datasets we have divided into five folds. Figure 6b shows a graph based on the results of the five classes.

The experimental dataset was maintained the same for each of these comparative experiments. For experiment 1, the FCNN approach achieved ultimate accuracy of 100%, 89%, and 97% for the proposed dataset, UCSD dataset, and JHU-CROWD dataset, respectively, using the proposed dataset, UCSD dataset, and JHU-CROWD dataset. Table 3, Table 4 and Table 5 shows the average microprecision, microrecall, and microF1 score of the suggested technique. All of these assessment matrices were created using the equations from [34]. For the proposed dataset, the average microaccuracy, microrecall, and microF1 score are 100%, 100% and 100%, respectively, compared to 90%, 89%, and 90% and 95%, 95% and 95% for the UCSD and JHU-CROWD datasets. The proposed FCNN model outperforms in terms of overall accuracy when using a state-of-the-art dataset that includes JHU-CROWD and UCSD. Furthermore, to the best of our knowledge, our suggested dataset is the only one of its kind in this field. These final accuracy comparisons shown at the Table 6.

The data augmentation applied in this Figure 6a with the five classes and got the augmentation verylow, low, medium, high, vhigh those classes. For getting five classes data augmentation we have use few technique. The techniques are below,

1. Pass a batch of images through our data augmentation pipeline
2. Return the augmented images
3. Randomly flip the images horizontally, randomly flip the images
4. Vertically, and rotate the images by 90 degrees in the counter

Figure 6b clearly shows that, from 0 to 50 epochs, there is no considerable change in the accuracy, whereas from 10 to 50 epochs, we observed no loss of data. However, for 50 to 100 epochs, the accuracy continued to increase. Finally, the accuracy for 100 epochs was got accuracy 100%. We have clearly seen that from 0 to 20 epochs there is slowly taking place loss of data, whereas from 20 to 80 and 80 to 100 epochs, there is a still slowly loss of data. Finally, the data loss at 100 epochs is 0.0023. On the other hand, the train loss and Val-loss; from 0 to 40 epochs, there is a slow change in the val-loss and at the same time no considerable change in the train loss. However, for 40 to 80 epochs and 80 to 100 epochs, it slowly changed with the Train loss as well as 40 to 80 and 80 to 100 epochs, with no considerable change in the val-loss. Finally, the train loss was 0.0023 and the val-loss was 0.99.

### 5.2. Experiment 2 (VGGNet)

For the experiment 2, the VGGNet technique obtained final accuracy of 98%, 97% and 97% for the proposed dataset, UCSD dataset, and JHU-CROWD dataset, respectively. Table 7, Table 8 and Table 9 illustrate the proposed method’s average microprecision, microrecall, and microF1 score. The formulae from [34] were used to create all of these evaluation matrices. The average micro precision, micro recall, and micro F1 score for the proposed technique is 95%, 95% and 95% for the proposed dataset, respectively, compared to 97%, 97% and 97% and 95%, 95% and 95% for the UCSD and JHU-CROWD datasets. Considering the use of a state-of-the-art dataset that includes JHU-CROWD and UCSD, the proposed FCNN model outperforms in terms of overall accuracy. Additionally, to the best of our knowledge, our proposed dataset is a unique dataset in this domain. These final accuracy comparisons shown at the Table 10.

### 5.3. Experiment 3 (ResNet50)

For the experiment 3, the ResNet50 technique obtained final accuracy of 99%, 91% and 97% for the proposed dataset, UCSD dataset, and JHU-CROWD dataset, respectively. Table 11, Table 12 and Table 13 illustrate the proposed method’s average microprecision, microrecall, and microF1 score. The formulae from [34] were used to create all of these evaluation matrices. The average micro precision, micro recall, and micro F1 score for the proposed technique is 100%, 100% and 100% for the proposed dataset, respectively, compared to 90%, 90% and 90% and 96%, 96% and 96% for the UCSD and JHU-CROWD datasets. Considering the use of a state-of-the-art dataset that includes JHU-CROWD and UCSD, the proposed FCNN model outperforms in terms of overall accuracy. Additionally, to the best of our knowledge, our proposed dataset is a unique dataset in this domain. These final accuracy comparisons shown at the Table 14.

## 6. Conclusions

In this paper, we use a DHCDCNNet model for crowd density analysis interpretation to explain our findings. With the multi-class structure of dilated convolution, it is possible to collect context at many scales. On two commonly used benchmark datasets for crowd analysis, we compared DHCDCNNet to current state-of-the-art techniques. We used two public datasets to execute our proposed method: the JHU-CROWD dataset, the UCSD dataset and proposed Hajj-Crowd dataset. In the experiments, the results showed that DHCDCNNet outperformed the state-of-the-art methods in terms of accuracy. By using our proposed DHCDCNNet method we can not solve the real time crowd analysis problem. In the future, we want to improve our DHCDCNNet approach to learn more about crowd behavior analysis. Action recognition algorithms can be used in conjunction with temporal information.

## Figures and Tables

**Table 1:** The proposed DHCDCNNet structure.

Layer Type	Output Shape	Param
conv2d (Conv2D)	(None, 118, 118, 32)	896
max_pooling2d (MaxPooling2D)	(None, 59, 59, 32)	0
conv2d_1 (Conv2D)	(None, 57, 57, 16)	4624
max_pooling2d_1 (MaxPooling2	(None, 28, 28, 16)	0
dropout (Dropout)	(None, 28, 28, 16)	0
flatten (Flatten)	(None, 12544)	0
dense (Dense)	(None, 128)	1,605,760
dropout_1 (Dropout)	(None, 128)	0
dropout_2 (Dropout)	(None, 128)	0
dense_1 (Dense)	(None, 50)	6450
dense_2 (Dense)	(None, 5)	255
Total params:	N/A	1,617,985
Trainable params:	N/A	1,617,985

**Table 2:** Comparison with state-of-the-art public datasets.

Dataset	No. of Image	Resolution
UCSD [13]	2500	158 × 238
UCF-CC-50 [7]	50	2101 × 2888
Mall [31]	2000	480 × 640
WorldExpo’10 [32]	3980	576 × 720
ShanghaiTech Part A [6]	482	589 × 868
ShanghaiTech Part B [6]	716	768 × 1024
JHU-CROWD [32]	4250	1450 × 900
NWPU-Crowd [33]	5109	2311 × 3383
PROPOSED HAJJ-CROWD	27,000	1914 × 922

**Table 3:** Result for the five classes proposed Hajj-Crowd dataset using FCNN Model.

Class	Precision	Recall	f1-Score	Support
VLOW	1.00	1.00	1.00	1080
LOW	1.00	1.00	1.00	1080
MEDIUM	1.00	1.00	1.00	1080
HIGH	1.00	1.00	1.00	1080
VHIGH	1.00	1.00	1.00	1080
micro avg	1.00	1.00	1.00	5400
macro avg	1.00	1.00	1.00	5400
avg	1.00	1.00	1.00	5400

**Table 4:** Result for the five classes UCSD dataset using FCNN Model.

Class	Precision	Recall	f1-Score	Support
VLOW	1.00	1.00	1.00	100
LOW	1.00	0.46	0.63	100
MEDIUM	1.00	1.00	1.00	100
HIGH	1.00	1.00	1.00	100
VHIGH	0.67	1.00	0.80	100
micro avg	0.90	0.89	0.90	500
macro avg	0.93	0.89	0.89	500

**Table 5:** Result for the five classes JHU-CROWD dataset using FCNN Model.

Class	Precision	Recall	f1-Score	Support
VLOW	1.00	1.00	1.00	160
LOW	0.81	1.00	0.89	160
MEDIUM	1.00	0.76	0.86	160
HIGH	1.00	1.00	1.00	160
VHIGH	1.00	1.00	1.00	160
micro avg	0.95	0.95	0.95	800
macro avg	0.96	0.95	0.95	800

**Table 6:** Comparision with the state-of-the-art dataset and proposed DHCDCNNet model.

Exp.	Dataset	Model	Final Accuracy
1	Hajj-Crowd	DHCDCNNet	100%
1	UCSD	DHCDCNNet	89%
1	JHU-CROWD	DHCDCNNet	97%

**Table 7:** Result for the five classes Hajj-Crowd dataset using VGGNet Model.

Class	Precision	Recall	f1-Score	Support
VLOW	1.00	1.00	1.00	1080
LOW	0.82	1.00	0.88	1080
MEDIUM	1.00	1.00	1.00	1080
HIGH	1.00	1.00	1.00	1080
VHIGH	1.00	1.00	1.00	1080
micro avg	0.95	0.95	0.95	5400
macro avg	0.96	0.95	0.95	5400

**Table 8:** Result for the five classes UCSD dataset using VGGNet Model.

Class	Precision	Recall	f1-Score	Support
VLOW	1.00	1.00	1.00	100
LOW	0.88	1.00	0.93	100
MEDIUM	1.00	0.86	0.92	100
HIGH	1.00	1.00	1.00	100
VHIGH	1.00	1.00	1.00	100
micro avg	0.97	0.97	0.97	500
macro avg	0.98	0.97	0.97	500

**Table 9:** Result for the five classes JHU-CROWD dataset using VGGNet Model.

Class	Precision	Recall	f1-Score	Support
VLOW	1.00	1.00	1.00	160
LOW	0.81	1.00	0.89	160
MEDIUM	1.00	0.76	0.86	160
HIGH	1.00	1.00	1.00	160
VHIGH	1.00	1.00	1.00	160
micro avg	0.95	0.95	0.95	800
macro avg	0.96	0.95	0.95	800

**Table 10:** Comparision with the state-of-the-art dataset and VGGNet.

Exp.	Dataset	Model	Final Accuracy
2	Hajj-Crowd	VGGNet	98%
2	UCSD	VGGNet	97%
2	JHU-CROWD	VGGNet	97%

**Table 11:** Result for the five classes Hajj-Crowd dataset using ResNet50 Model.

Class	Precision	Recall	f1-Score	Support
VLOW	1.00	1.00	1.00	1080
LOW	0.99	0.99	1.00	1080
MEDIUM	1.00	1.00	1.00	1080
HIGH	1.00	1.00	1.00	1080
VHIGH	1.00	1.00	0.99	1080
micro avg	1.00	1.00	1.00	5400
macro avg	1.00	1.00	1.00	5400

**Table 12:** Result for the five classes UCSD dataset using ResNet50 Model.

Class	Precision	Recall	f1-Score	Support
VLOW	0.97	0.97	0.97	100
LOW	0.86	0.75	0.80	100
MEDIUM	0.86	0.95	0.90	100
HIGH	0.98	0.98	0.97	100
VHIGH	0.86	0.80	0.75	100
micro avg	0.90	0.90	0.90	500
macro avg	0.91	0.91	0.91	500

**Table 13:** Result for the five classes JHU-CROWD dataset using ResNet50 Model.

Class	Precision	Recall	f1-Score	Support
VLOW	1.00	1.00	1.00	160
LOW	0.81	1.00	0.89	160
MEDIUM	0.97	0.97	0.97	160
HIGH	0.97	1.00	0.94	160
VHIGH	1.00	095	097	160
micro avg	0.96	0.96	0.96	800
macro avg	0.96	0.96	0.96	800

**Table 14:** Comparison with the state-of-the-art dataset and ResNet50.

Exp.	Dataset	Model	Final Accuracy
3	Hajj-Crowd	ResNet50	99%
3	UCSD	ResNet50	91%
3	JHU-CROWD	ResNet50	97%

## Data Availability

In this section, we provided the dataset and last accessed the link of date (Dated: 8 March 2022) https://github.com/romanbhuiyan/Code-and-Hajj-Crowd-dataset-2021/blob/main/Dataset. Code (Dated: 8 March 2022) https://github.com/romanbhuiyan/Hajj-Crowd_Augmented_Code.
